# Linking social and built environmental factors to the health of public housing residents: a focus group study

**DOI:** 10.1186/s12889-015-1710-9

**Published:** 2015-04-10

**Authors:** Erin Hayward, Chidinma Ibe, Jeffery Hunter Young, Karthya Potti, Paul Jones, Craig Evan Pollack, Kimberly A Gudzune

**Affiliations:** The Johns Hopkins University School of Medicine, Baltimore, MD USA; Johns Hopkins Health Care, LLC, Baltimore, MD USA; Department of Health Policy and Management, The Johns Hopkins Bloomberg School of Public Health, Baltimore, MD USA; Welch Center for Prevention, Epidemiology, and Clinical Research, Johns Hopkins Medical Institutions, Baltimore, MD USA; Department of Epidemiology, The Johns Hopkins Bloomberg School of Public Health, Baltimore, MD USA; University of Maryland School of Medicine, Baltimore, MD USA; Division of General Internal Medicine, Department of Medicine, Johns Hopkins University, 2024 E. Monument St, Room 2-621, Baltimore, MD 21287 USA

**Keywords:** Urban health, Public housing, Social determinants of health, Environmental health

## Abstract

**Background:**

Public housing residents have a high risk of chronic disease, which may be related to neighborhood environmental factors. Our objective was to understand how public housing residents perceive that the social and built environments might influence their health and wellbeing.

**Methods:**

We conducted focus groups of residents from a low-income public housing community in Baltimore, MD to assess their perceptions of health and neighborhood attributes, resources, and social structure. Focus groups were audio-recorded and transcribed verbatim. Two investigators independently coded transcripts for thematic content using editing style analysis technique.

**Results:**

Twenty-eight residents participated in six focus groups. All were African American and the majority were women. Most had lived in public housing for more than 5 years. We identified four themes: public housing’s unhealthy physical environment limits health and wellbeing, the city environment limits opportunities for healthy lifestyle choices, lack of trust in relationships contributes to social isolation, and increased neighborhood social capital could improve wellbeing.

**Conclusions:**

Changes in housing and city policies might lead to improved environmental health conditions for public housing residents. Policymakers and researchers may consider promoting community cohesiveness to attempt to empower residents in facilitating neighborhood change.

## Background

Currently, 1.2 million Americans are estimated to reside in federally subsidized public housing units [1]. Studies have shown that nearly 50% of adults living in public housing are obese [2-4] and have nearly two-fold greater risk of hypertension compared to residents in the same city, even with adjustment for socioeconomic factors [4]. The health problems faced by public housing residents may be due, in part, to the high poverty, urban environments where public housing is often situated [5]. The importance of neighborhood environments for public housing residents was underscored by the long-term results of the United States Department of Housing and Urban Development (HUD)-sponsored Moving to Opportunity for Fair Housing Demonstration (MTO) in which public housing residents were randomized to remain in public housing, receive a voucher to move (without restrictions), or receive a voucher to move to a low poverty neighborhood. Public housing residents who had the opportunity to move to a neighborhood of higher socioeconomic status (SES) had a reduced prevalence of extreme obesity and diabetes as compared to public housing residents who did not have this opportunity [4]. The mechanisms through which low neighborhood SES impacts the health of public housing residents remain poorly understood.

Both the social and built environments may be two important mechanisms that shape the health of public housing residents. For this study, we considered the social environment to include neighborhood social structure and interpersonal relationships. In other low SES communities, social capital may influence the spread of health information and shape ideas and behaviors that can positively or negatively impact residents’ health [6]. A community’s built environment may also influence behaviors and health outcomes. For example, obesity has been linked to limited availability of grocery stores and recreation areas and increased exposure to crime and traffic [7-9]. Understanding how dimensions of the social and built environments influence public housing residents’ health could provide insight on how to best tailor interventions to this high-risk population.

Our objective was to understand how public housing’s social and built environments might influence residents’ health and wellbeing. We conducted focus groups of public housing residents living in a low SES community to understand their perceptions of these factors and their relationship to health.

## Methods

**Figure 1:**
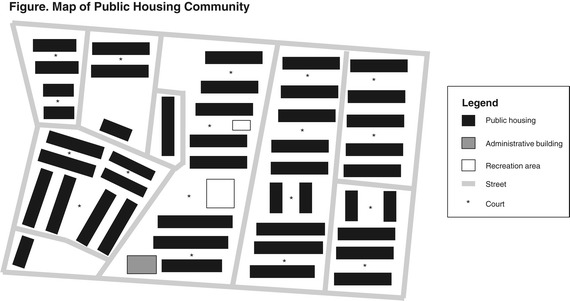
**Map of public housing community showing locations of buildings and housing courts.**

From July to November 2011, we conducted focus groups of residents from a public housing community in Baltimore, MD. This community is a clustered public housing development that has 700 units located in 45 two-story buildings with over 95% occupancy. Most low-rise buildings form courts where buildings’ front entrances open onto common pedestrian areas (Figure 1). The city housing authority manages this property and residents elect a local tenant council to represent their interests. The surrounding neighborhood consists of single-family row homes, many of which are derelict or vacant. This community is located in a census tract where 27% of families live in poverty and violent crime rates, including homicides and non-fatal shootings, are high [10]. We elected to use volunteers from this community, as public housing residents can be a hard-to-reach population. We recruited participants by distributing flyers in the development and through in-person recruitment during monthly resident meetings by study investigators (CI, KG).

We used focus groups to generate discussion on topics among community members. Each focus group was conducted onsite, included between 4 and 6 participants, and lasted approximately 90 minutes. All participants completed a questionnaire to obtain demographic information. Participants received a $25 Visa gift card as compensation for their time. Based upon the World Bank’s approach to qualitative research for evaluating community social capital [6], we developed a semi-structured guide including open-ended questions and reflective probes covering the themes of health, neighborhood resources, positive and negative attributes of the community, neighborhood social structure, and interpersonal relationships (Table 1). We pilot tested this guide for comprehension and understanding among local community health workers who were racially and socioeconomically concordant with participants.

**Table 1:** **Content of semi-structured guide**

1	Tell us who you are, and how long you’ve lived in East Baltimore.
2	I would like to better understand how you view your community. We are going to make a map of your community.
	Probe 1: What do you consider the boundaries of your neighborhood?
	Probe 2: What are the important resources in your neighborhood? Where are they located? Why do you choose these over others?
	Probe 3: Who are the important people in your community? What do they do and where are they located? Are their important people who do not have “official titles” in the community?
	Probe 4: What do you do for fun? Where?
3	Is there a strong sense of community where you live?
	Probe 1: Do neighbors look out for one another? Help each other out?
	Probe 2: How has the community worked together in the past to solve a problem? Or have members in the community ever helped you with a problem?
4	List 6 major problems in your community.
	Probe 1: How do you rank these problems in order of importance?
	Probe 2: Where does health rank?
5	How does information spread through your community? For example, if there was a free give-away on one side of the neighborhood, how would you let your friends know about it?
	Probe 1: Person-to-person? Phone? Cell phone? Internet? Email? Flyers? Mail?
6	We would like to better understand who are the important people in your life. I am handing out charts* for you to complete about these relationships. Please think about each person listed in the left column and what role they play in your life. If you have more than one person in one of the categories, then please think about the person who you trust the most or who gives you the most support. In the top row are listed different types of situations or activities. Please place an “X” in a box, if that person plays that role in your life. Place an “X” in all boxes that apply for each person, unless that person does not exist or is not involved in your life; then, only mark “not applicable” or “not in my life.” For example, if your sister was your main confidant and support system, then you might place an “X” in the boxes “trust with a secret” and “turn to if someone close to me died.” If you have any questions or need any help completing this chart, please ask the assistant moderator or me for assistance. Looking at the chart you just completed, what do you notice about the different people who provide support in your life?
	Probe 1: What do the people who provide you support have in common?
	Probe 2: What do the people who do NOT provide you support have in common?

*Exchange matrix charts available from authors upon request.

A moderator (CI) used this guide during all groups and an assistant moderator (KG) recorded field notes. Given her doctorate in health behavior and research focus on communities, CI was selected to moderate the groups. KG is a clinician investigator (no participants in these groups were her patients). At the beginning of each group, the moderator read the informed consent and participants asked questions. Each participant gave oral consent. False names were used during the groups to ensure anonymity. The moderator discussed the confidential nature of the group before beginning with the questions. The Institutional Review Board of The Johns Hopkins University School of Medicine approved this study. Immediately following each focus group, the moderator and assistant moderator had a debriefing session to discuss overarching themes of the group as compared to prior groups. Once similar ideas had been expressed within three groups, we determined that we had reached thematic saturation and halted data collection. We achieved thematic saturation after 6 focus groups.

Each group was audio recorded and transcribed verbatim. We used Atlas.ti 6.2 qualitative software to facilitate data management and analysis. Given the exploratory nature of this study, we used an inductive thematic analysis approach. To identify meaningful portions of the text and generate codes, we used an editing-style analysis technique [11]. We used the first transcript to develop a codebook, which was then applied and expanded as new concepts were identified during the analysis of the remaining groups. A separate team of investigators led the qualitative analysis (EH (medical student), KP (undergraduate student), and PJ (research assistant from the community)). Two reviewers (EH, KP, or PJ) independently coded each transcript and met regularly to apply the final codes to the transcripts. An additional investigator was available (KG) to resolve conflicts, although none arose. Quotations and final codes were discussed between study investigators (EH, KP, PJ, KG) and organized into conceptual themes using a consensus process. The other study team members (CI, JY, CP) reviewed this work and had the opportunity to contest the themes and subthemes.

## Results

Twenty-eight residents participated in six focus groups. All were African American and the majority were women. Participants’ mean age was 50.6 years (SD 12.3; range 21–71). Eighty-six percent of participants reported living in Baltimore their entire lives, and 55% had lived in public housing ≥5 years with a mean of 8 years (SD 6). Seventy-nine percent held a high school diploma or equivalent.

We identified four themes related to the social and physical environment experienced by public housing residents (Table 2). Below, we present each of these themes along with representative quotes from each subtheme to demonstrate how participants perceived these factors to influence their health and wellbeing.

**Table 2:** **Themes and subthemes**

**Theme 1**	**Public housing’s unhealthy physical environment limits health and wellbeing (5)**
	1A: Neighbors’ actions create poor sanitary conditions that have negative health effects (5)
	1B: Housing Authority practices contribute to unsafe community conditions (5)
**Theme 2**	**City environment limits opportunities for healthy lifestyle choices (4)**
	2A: Limited access to recreation facilities due to city’s high prices and facility closures (4)
	2B: High local crime prevents residents from using neighborhood outdoor spaces for recreation (4)
**Theme 3**	**Limited trustworthy interpersonal relationships contribute to social isolation (5)**
	3A: Trusted contacts usually limited to select family members (4)
	3B: Individuals weigh social isolation versus risk of gossip when engaging with friends and neighbors (5)
**Theme 4**	**Increased neighborhood social capital could improve wellbeing (5)**
	4A: Social ties between neighbors vary by geographic clusters (4)
	4B: Community lacks unified voice to increase social cohesion and advocate for change (4)
	4C: Increased neighborhood collective action could improve community conditions (3)

Numbers in parentheses represent the number of groups in which each theme and subtheme was discussed.

### Theme 1: Public housing’s unhealthy physical environment limits health and wellbeing

Environmental health was a significant concern for participants, who listed numerous problems including rats, trash, and safety, as negatively influencing the health and wellbeing of community members (subtheme 1A). Poor sanitation was cited as one of the community’s biggest problems. Participants often blamed other residents for creating unsanitary conditions, which then promoted rat infestation. *“We have to try to get rid of some of the rats…if some of the people don’t throw a lot of stuff out on the street or in the courts and stuff, it won’t be so bad.”* Participants noted that these conditions also existed inside their neighbors’ homes. “*You can look up their steps and see all the trash… you gonna always have a mouse as long as those neighbors are like that because it takes the whole row [of housing units] to be clean.”* Many residents felt that the city housing authority failed to enforce lease terms that penalized these behaviors, and therefore, contributed to the continued problems. Participants identified a connection between these conditions and negative health impacts. “*People around [here], they don’t care about their health. Hey, I care about mine. I breathe in all that debris coming from outside, that trash…the rats, all kind of bugs.”* Participants were particularly concerned about the effects of the unsanitary environment on residents with asthma or other respiratory conditions.

Participants perceived a lack of investment by the city housing authority to improve living conditions (subtheme 1B). Participants reported that problems in their homes would remain unaddressed by staff, resulting in the persistence of unsafe living conditions including electrical and heating/cooling issues. “*Every summer we have the problem of our lights going out, and they promise and promise they gonna fix it. The problem is they need to rewire it, but they won’t.”* In addition, participants perceived that certain housing policies contributed to hazardous living conditions for residents, especially children. *“[The city housing authority] say you can’t have more than one air conditioner… They don’t understand that people’s kids have asthma, and it’s hot… Like you just got in your car hot, and they don’t care.”*

### Theme 2: Neighborhood environment limits opportunities for healthy lifestyle choices

Participants reported that several aspects of the neighborhood surrounding the public housing facility negatively affected their lives and ability to make healthy choices. Four groups expressed concern over of the limited availability of recreation facilities to local children and adults, due to the city setting high access fees and the planned closing of some facilities (subtheme 2A). “*My daughter was gonna put [my grandchildren in a program at the recreation center], but you actually have a fee to pay to be on the team. It's an extremely high [fee] to be a low-income community.”*

Participants felt that high local crime rates created an unsafe environment (subtheme 2B), especially at the community’s playground. As a result, caregivers often limited outdoor activity for children. *“The kids could be up out there playing on the playground…then you got the police chasing people… That’s where I think that [the children] see too much that they ain’t supposed to see.”* Participants consistently reported that drugs and crime had profound negative effects on their community. *“[Dealers] trying to make your block a drug block and sell drugs. They laugh at older people [that] are scared to come outside.”* Many participants expressed a desire for a more consistent police presence in the neighborhood to prevent crime and drug activity, rather than only coming to the community during raids or when emergency services were called. *“If something serious happened…you see the police walk through a couple of times and a couple of days later they’re gone…what we need is to have them to walk through just about every day.”*

### Theme 3: Lack of trust in relationships contributes to social isolation

Participants usually identified family members as trustworthy, rather than friends (subtheme 3A). *“If I need help, I'll ask my family only.”* Several participants noted that they had complete trust in only one individual in their family, as other members were either deceased or perceived as unreliable, often due to substance abuse. While participants indicated that they often spent time with a significant other, many comments described tenuous relationships. *“I can’t tell my husband a lot of secrets… He’ll go out there and tell everything.”*

Participants reported mixed feelings about their friends and neighbors. Some cited occasions during which their neighbors helped them in difficult times*. “I lost a son and didn’t have no insurance, so the community got involved and basically paid for my son’s funeral. And there was still money left over to help take care of me and my family.”* However, other participants noted that trusting friendships were limited due to fear of gossiping. *“I don't trust friends, because they talk to you and then they talk to the next neighbor, and they talk to them about you. So I try to stay away from that*.” Skepticism about neighbors also arose because some individuals tended to only share information about local events or giveaways after they had first benefited themselves. “*…I have a neighbor that knows about everything, so she’ll let you know. But, once again, she’s one of the ones that will go and get what they have free and tell you once she get back, right, and then any time you get there - It’s gone.*” Overall, a lack of trust in many social network members seemed to promote social isolation (subtheme 3B). Statements such as “*I don't have any friends and my sister lives in South Carolina… So [it’s me] and my cats” or* “*I'm a loner”* were common.

### Theme 4: Increased neighborhood social capital could improve wellbeing

The quality of social ties among neighbors seemed to vary by geographic location within the community (subtheme 4A), which is divided into courts. Residents of more close-knit courts experienced times of community cohesion, such as when residents worked together to eradicate rats or supported a family after the death of a loved one. In these courts, older residents tended to look out for the wellbeing of children through the windows or from the front steps of their homes. Conversely, residents of less close-knit courts often expressed negative feelings about their neighbors. *“People in the community are so busy trying to [get] down on each other when really basically none of us is better than the next one.”* Participants in three groups noted that solidarity developed among individuals who had been living in the community for several years. *“The residents that’s been here the longest, I think we stick together pretty good, even through our ups and downs*.” These close ties do not necessarily extend to new neighbors, who may be perceived as outsiders, and therefore distrusted. These new residents described being wary of enforcing informal social control: *“I see folk do stuff sometimes, and I wanna say something, but it’s just not that type of environment where you can just speak up to the individual.”*

Participants perceived that the community lacked a unified voice to bring about social cohesion and advocate for change (subtheme 4B). While the community has resident-elected members to serve on a council, few participants viewed this group as effectively making changes. *“There's no leadership as far as a president of the community.”* The participants were divided on the resident council’s leadership. Some expressed how these leaders made promises to improve the community, but they had yet to see change. Others admired these local leaders and felt that the lack of changes was related to residents’ general non-participation in community meetings and events. *“…folks should attend the meetings and stand up and be counted….that way we can get things done as a big group.”*

Many participants expressed a desire for more collective action as a means to improve quality of life in the community (subtheme 4C). *“It would be more safe for the community…it will basically make us look good as residents ‘cause we came as one trying to keep it together”.* Another group noted that if residents invested more in the community, they could attract new local resources that could improve their health. “*[Residents should] take pride in your stuff … it brings the quality of life up anyway. Then folk, at like a big chain grocery store that you wouldn’t normally find in the city or in the county, they wouldn’t mind moving not too far from here or around the corner.”* Overall, participants perceived limited collective action or social cohesion currently, but some participants described themselves as part of a small contingent willing to work to improve community conditions. *“[We] battle about keeping the block clean, because I’m telling people [that the housing authority] not gonna do nothing, so we gotta do it ourselves.”* These participants expressed a belief that collective action to keep the neighborhood clean would lead to increased informal social control to ensure these healthy conditions persisted. Efforts by the housing authority to employ workers to clean up the community also drew participants’ ire. Some perceived this program as a missed opportunity for collective action among residents. *“Folks throw trash because they know in the morning there’s people that come around here and clean up.”* Others perceived hiring outside labor as an expenditure of resources that would be better kept in the community. “*Years ago you could clean up your own neighborhood and they might take X amount of dollars off of the rent.”*

## Discussion

Overall, our results describe a complex picture of how the physical and social environment impacts the health of public housing residents. Four themes emerged from our focus group discussions: the physical environment of public housing limits health and wellbeing of its residents, neighborhood environment limits opportunities for healthy lifestyle choices, lack of trust in relationships contributes to social isolation, and increased neighborhood social capital could improve wellbeing. While there was general agreement among participants that characteristics of the physical environment—including rats and trash—adversely affected health, views towards the social environment were more mixed. Several participants expressed that they could envision a cleaner and safer community through improved social relations and collective action. These participants also believed that increased participation in neighborhood improvement efforts would demonstrate to outsiders, including city officials and business owners, that their community merits investment. However, these goals may be difficult to achieve as many identified their neighbors’ current actions as the primary cause of local sanitation problems and community members lack of trust and openness among one another. This mixed-picture of the social environment highlights both the opportunities and challenges for public housing residents in improving health.

### Influence of built environment factors on public housing residents’ health

The absence or presence of environmental pollutants, general cleanliness of the community, and civic engagement have been key determinants in perception of a community as healthy or unhealthy [12]. Residents in our study perceived a connection between unsanitary and hazardous home conditions with negative health consequences, especially respiratory health. Their perceptions align with findings from a prior study of building characteristics and indoor allergens that found cockroach and mouse allergens were more likely to be present in public housing units than other types housing [13]. Similarly, another study found that children living in public housing had a significantly increased risk of asthma as compared to those living in private family homes. In this study, self-reported presence of cockroaches and rats were linked with current asthma [14]. We build upon these prior studies among public housing residents by identifying factors perceived to exacerbate these conditions, including housing authority enforcement of lease terms and the behaviors of other residents. Changes in property management practices, such as improving maintenance and pest control or creating leasing agreements that regulate home and neighborhood sanitation, may improve environmental conditions and health.

In addition to the home environment, residents believed that the surrounding neighborhood environment limited their opportunities to make healthy lifestyle choices. Specifically, focus group participants reported that recreation center fees often limited their access and their children’s access to these facilities and that local crime prevented them from using outdoor spaces around their homes. McAlexander and colleagues observed that public housing residents with greater access to physical activity resources had decreased body fat [15]. These researchers hypothesized that residents would be deterred from using sidewalks for exercise or to access physical activity resources if they lived in neighborhoods where the majority of crimes took place on streets or sidewalks. In general, neighborhood safety has been associated with physical and mental health [12], and a prior study found that low-income residents perceived that threats to their safety were coming from others within their local area [16]. Yet, the built environment can aid crime reduction and improved quality of life by selecting certain design strategies. This concept – Crime Prevention through Environmental Design (CPTED) – promotes higher density neighborhoods, surveillance, mixed-use development, and elimination of derelict or vacant buildings to reduce the opportunities for crime to occur [17]. Our results suggest that changes in city policies such as lower recreation center fees and routine police presence in these neighborhoods may increase residents’ engagement in exercise and recreation activities. In addition, though we recognize that substantial fiscal barriers are likely to exist, the city may need to consider employing some CPTED strategies like eliminating the nearby vacant row homes and promoting more businesses to come into the area to create a more mixed-use neighborhood.

### Influence of social environment factors on public housing residents’ health

Several participants in our focus groups described feeling socially isolated, due in part to lack of trust in interpersonal relationships. This may be an additional factor contributing to poor health, as social isolation has been linked with increased risk of death and chronic disease, including cardiovascular disease [18]. However, our results contrast with prior studies of public housing residents. Keene and Geronimus found that public housing residents were significantly more likely to report a supportive neighborhood environment where neighbors rely on one another and watch out for each other’s children [19]. Another study observed that Atlanta public housing residents had a sense of community attachment that was linked to collective efficacy and social support [20]. Increased social cohesion has been associated with increased perceived safety in a community [21]. A prior study showed that residents of a less cohesive and safe low-income neighborhood had significantly worse self-rated health than residents of other neighborhoods [16]. We suspect that the lack of trust in neighbors, which was commonly reported among our participants, may adversely affect community attachment in public housing neighborhoods. Additional studies are needed to determine whether or not social isolation occurs commonly among public housing residents, and if so, the factors that contribute to its development, as this could be an important target for future interventions.

### Interaction between built and social environmental factors

Several subthemes that we identified highlight how the built and social environments may influence one another. Many residents identified unsanitary conditions within the physical environment as negatively contributing to health; however, these conditions occurred due to the actions of other community members. Social control has been identified as a powerful influence to regulate these behaviors [22]. Many residents did not feel empowered to talk to their neighbors about not littering or keeping a clean house, and therefore, social regulation may not develop. In addition, we noted that residents in different courts display different relationships, which might suggest that how the neighborhood is designed might have an effect on sense of community. For example, a prior study in Atlanta found that communities with more walkable site designs (higher commercial floor space to land area ratio) and urban designs with pedestrian-friendly commercial areas were associated with a greater sense of community [23]. In this study, we are unable to explore possible environmental design features of public housing courts with varying degrees of cohesiveness, as we did not obtain address information from participants due to confidentiality protections. We believe that future studies should consider obtaining this information in order to understand what design factors promote a more socially cohesive housing court.

Finally, the participants in our study believed that increased neighborhood social capital could facilitate improved environmental conditions that translate into increased wellbeing. A framework for implementing a social capital approach to promote health equity has been developed, which provides recommendations for policymakers, local practitioners, and communities [24]. In this framework, social capital is posited to aid communities in developing networks of support and social action, and as a result, gain economic and other resources. Our findings suggest that an effective strategy to improve neighborhood-level health may involve interventions to improve neighborhood environment by fostering social cohesion among community members. Social cohesion depends upon the buy-in and participation of the local community. Prior interventions that successfully promoted cohesion have led to positive impacts on health and lifestyle. One initiative increased exercise rates within a multicultural public housing development through the creation of social walking groups within the community [25]. Other successful interventions have focused on a community’s common values or priorities to address health outcomes. For example, one initiative improved social cohesiveness by uniting low-income community members around activities to promote their children’s wellbeing [26]. Improved social cohesiveness has been observed to be an important factor in environmental improvements, including lower levels of violence at the community level [27] and greater perceived safety in public housing [21]. Having a larger social network has been linked with increased physical activity among adults in public housing [28].

### Strengths and limitations

Our study has important strengths. Our sample was predominantly female, which is consistent with population statistics for public housing residents. We recruited participants with a wide age range who had lived in public housing for varying amounts of time to capture the diverse experiences of different populations. We used an iterative and consensus-driven process during coding to increase reliability. We had a diverse team – healthcare providers and a community member – to code text and generate themes and subthemes. Nonetheless, our study has several limitations, among them that all participants were recruited from a single housing development in Baltimore. Residents of other developments in Baltimore, or those in other cities that are managed by different housing authorities, may express different views. Our sample may represent the voices of more activated community members, as they responded to a flyer and were willing to participate in a group discussion. We were unable to explore possible environmental design features of public housing courts with varying degrees of cohesiveness. We limited our conceptualization of social environment to neighborhood social structure and interpersonal relationships, and did not focus on cultural factors. Despite these limitations, we believe this qualitative study provides deeper insights into residents’ perceptions of environmental factors that influence their health.

## Conclusions

Public housing residents perceive that community-level factors influence their health, and our focus group results may provide insight on potential changes in housing and city policies. The local housing authority could work to address some of the environmental factors such as poor sanitation and unsafe conditions to improve residents’ wellbeing while recognizing the complex interplay that exists between the social and physical environments for public housing residents. Our results suggest that engaging residents in future policy-making processes may increase their perception of individual empowerment and community collective action.
